# Presence of DNA from *Chlamydia*-like organisms in the nasal cavities of grey seal pups (*Halichoerus grypus*) and three different substrates present in a breeding colony

**DOI:** 10.1186/s12917-021-03032-3

**Published:** 2021-10-13

**Authors:** Mark P. Dagleish, Allen F. Flockhart, Johanna L. Baily, Ailsa J. Hall, T. Ian Simpson, David Longbottom

**Affiliations:** 1 Moredun Research Institute, Pentlands Science Park, Bush Loan, Penicuik, UK; 2 Present address: School of Applied Sciences, Edinburgh Napier University, Sighthill Campus, Edinburgh, UK; 3 Sea Mammal Research Unit, Scottish Oceans Institute, University of St Andrews, St Andrews, UK; 4 Present address: Institute of Aquaculture, University of Stirling, Stirling, UK; 5 Biomathematics and Statistics Scotland, JCMB, The King’s Buildings, Peter Guthrie Tait Road, Edinburgh, UK; 6 Present address: School of Informatics, University of Edinburgh, Crichton Street, Edinburgh, UK

**Keywords:** *Chlamydia*-like organisms, *Parachlamydiaceae*, *Rhabdochlamydiaceae*, *Simkaniaceae*, sentinel species, coastal reservoir

## Abstract

**Background:**

*Chlamydia*-like organisms (CLO) have been found to be present in many environmental niches, including human sewage and agricultural run-off, as well as in a number of aquatic species worldwide. Therefore, monitoring their presence in sentinel wildlife species may be useful in assessing the wider health of marine food webs in response to habitat loss, pollution and disease. We used nasal swabs from live (*n* = 42) and dead (*n* = 50) pre-weaned grey seal pups and samples of differing natal substrates (*n* = 8) from an off-shore island devoid of livestock and permanent human habitation to determine if CLO DNA is present in these mammals and to identify possible sources.

**Results:**

We recovered CLO DNA from 32/92 (34.7%) nasal swabs from both live (*n* = 17) and dead (*n* = 15) seal pups that clustered most closely with currently recognised species belonging to three chlamydial families: *Parachlamydiaceae* (*n* = 22), *Rhabdochlamydiaceae* (*n* = 6), and *Simkaniaceae* (*n* = 3). All DNA positive sediment samples (*n* = 7) clustered with the *Rhabdochlamydiaceae*. No difference was found in rates of recovery of CLO DNA in live versus dead pups suggesting the organisms are commensal but their potential as opportunistic secondary pathogens could not be determined.

**Conclusion:**

This is the first report of CLO DNA being found in marine mammals. This identification warrants further investigation in other seal populations around the coast of the UK and in other areas of the world to determine if this finding is unique or more common than shown by this data. Further investigation would also be warranted to determine if they are present as purely commensal organisms or whether they could also be opportunistic pathogens in seals, as well as to investigate possible sources of origin, including whether they originated as a result of anthropogenic impacts, including human waste and agricultural run-off.

## Background

Environmental contamination of coastal land and inshore waters is commonly associated with municipal waste, sewage and agricultural run-off, and several studies have shown that marine wildlife can become infected with bacterial organisms of terrestrial origin [1–3]. As a top-tier aquatic predator and inhabitant of inshore ecosystems shared by man, grey seals (*Halichoerus grypus*) can act as a potential environmental sentinel for humans and the wider health of marine food webs in response to habitat loss, pollution and disease [4]. *Chlamydia*-like organisms (CLO; also known as *Chlamydia*-related bacteria) are obligate intracellular bacteria that have been associated with pneumonia and adverse reproductive outcomes in humans [5–7] and have also been recognized as respiratory pathogens in fish affecting aquaculture around the globe [5, 8–10]. Studies have also shown an association of CLOs with free-living amoeba present in salt and fresh water [11, 12], suggesting that they may be a reservoir for transmission to other aquatic species. Other studies have revealed a high prevalence of CLO in ruminant species, particularly cattle, suggesting that they are ubiquitous in the environment and possibly act as commensals that may become abortigenic pathogens under certain conditions [13–18].

The aim of this study was to determine whether any CLO DNA could be detected in the nasal cavities of pre-weaned grey seal pups and their differing natal substrates in a breeding colony on an island (Isle of May; 56.18° N 2.55° W), just off the coast of Edinburgh, in Scotland, in the UK as a preliminary speculative investigation to see if CLOs are present. To this end, pre-existing samples that had been collected as part of a PhD project investigating pathology in this seal population were investigated in this study [19].

## Results

**Fig. 1:**
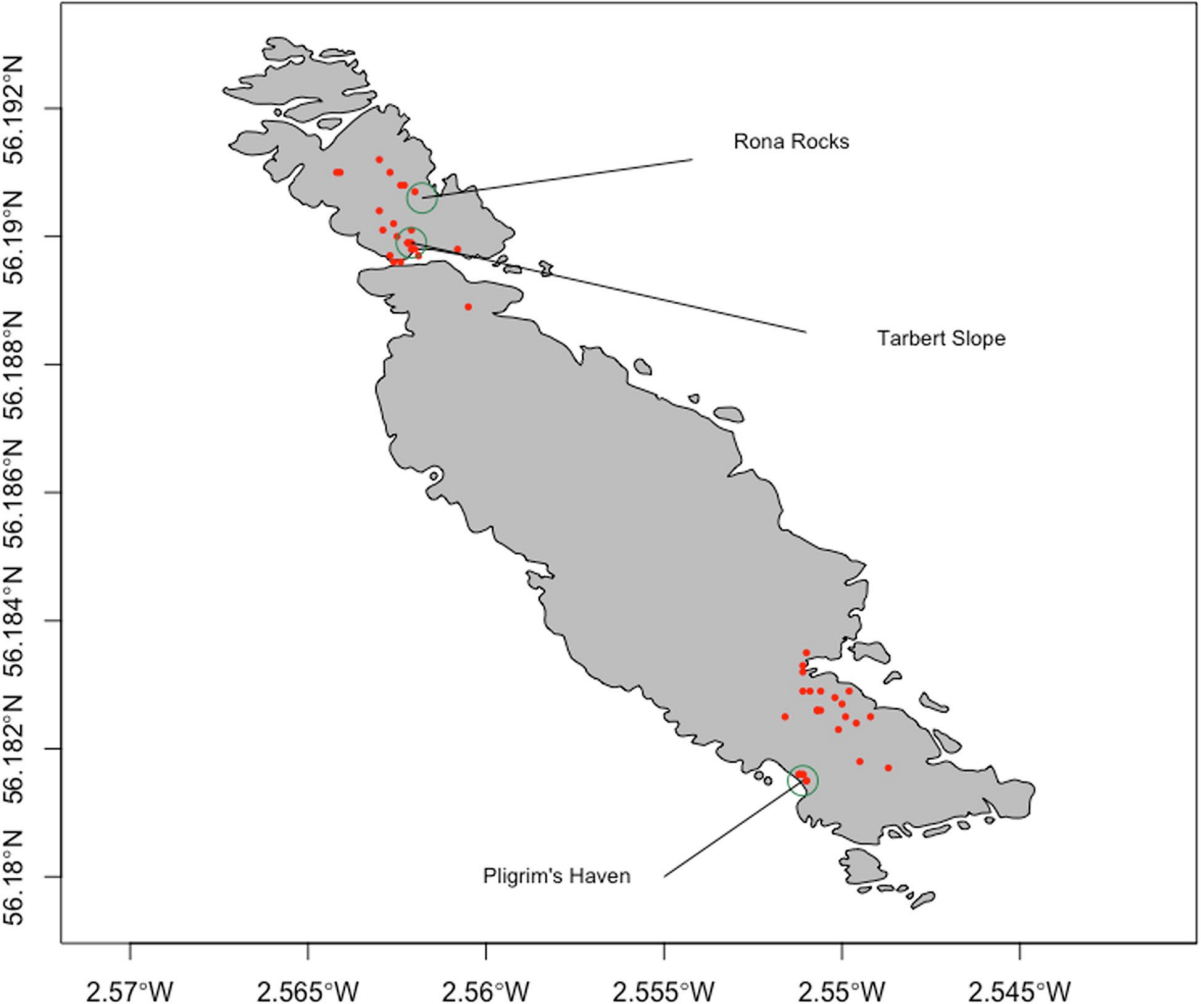
Map of Isle of May and location of seal breeding areas. Map showing the locations of the three main pupping sites (grey circles), where live grey seal pups (CL) were sampled, each comprised of a highly different substrate; a tidal boulder beach (Pilgrim’s Haven), a muddy grassy slope (Tarbet Slope) and rocky stagnant pools (Rona Rocks). Red circles represent locations where sampled dead grey seal pups were found. This illustration was computer generated using the R software environment for statistical computing and graphics [20]

In this study, PCR fragments of CLO DNA extracted from 92 nasal swab samples obtained from live (CL, *n* = 42) and dead (CD, *n* = 50) seal pups found on the island in the three main breeding areas were sequenced. These breeding areas were located in sites differing widely in substrate, which were also sampled, and DNA extracted for PCR analysis. The substrates sampled in the three areas were from tidal boulder beach at Pilgrim’s Haven (TBB, *n* = 3), a muddy grassy slope at Tarbet Slope (MGS, *n* = 3), and a rocky stagnant pool at Rona Rocks (RSP, *n* = 2) (Fig. 1). Good quality sequence information was obtained successfully for 32 (CL; *n* = 17, CD; *n* = 15) of the 92 (34.7%) nasal swab samples tested (sequences submitted to GenBank under accession numbers KT258813-KT258862) and for seven of the eight (77.7%) environmental sediment samples tested (GenBank accession numbers KT258863-KT258870); amplified products ranged in size from 261 bp to 277 bp. There was no significant difference in recovery rates of CLO DNA from nasal swabs from CL versus CD seal pups (*p* = 0.41).

**Fig. 2:**
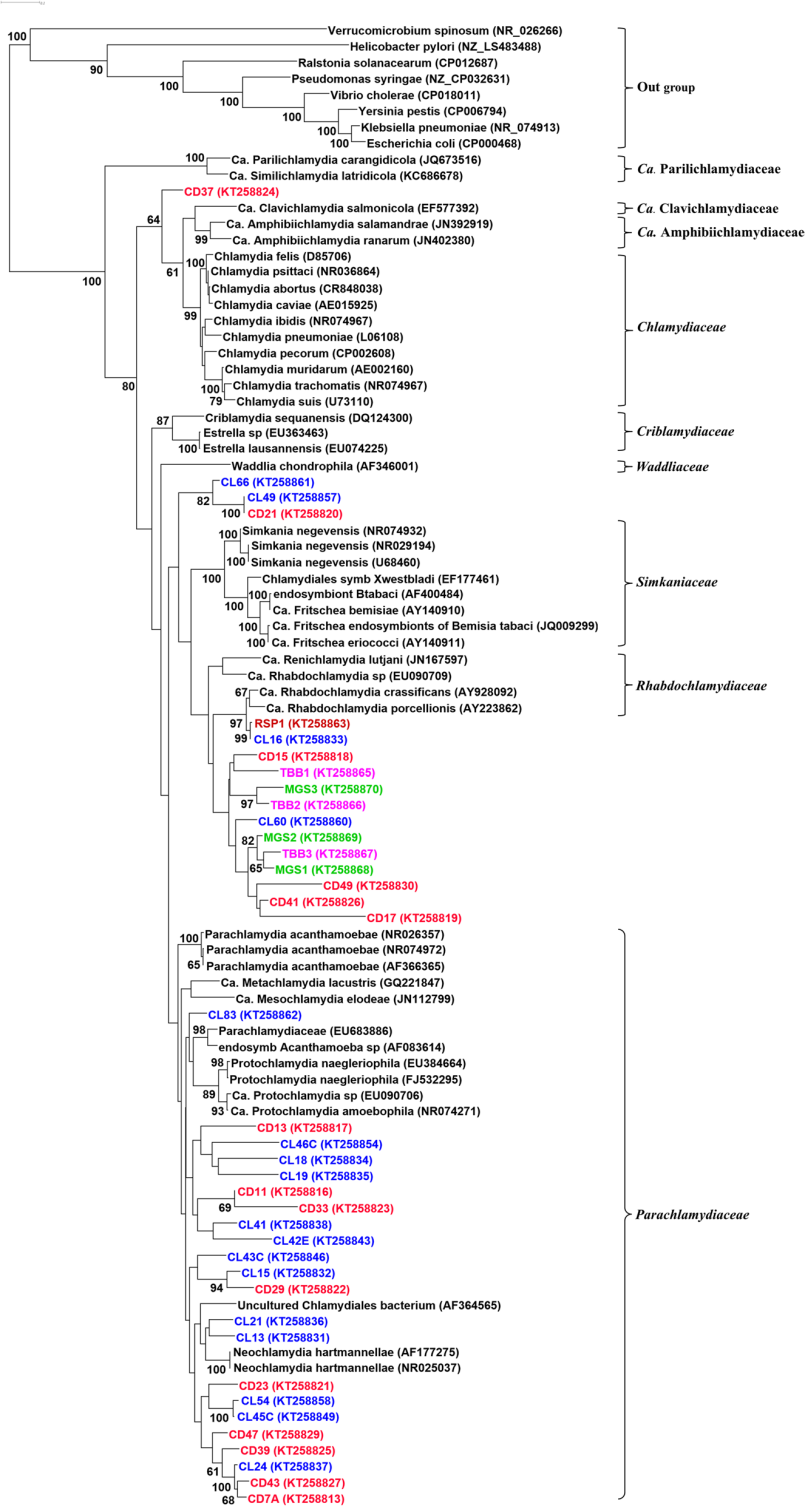
PhyML tree demonstrating the relationship between the DNA of chlamydial organisms isolated from the nasal cavities of live (CL, *n* = 17, in blue) and dead (CD, *n* = 15, in red) seal pups and three different associated substrates (tidal boulder beach [TBB, *n* = 3, in magenta], muddy grassy slope [MGS, *n* = 3, in green] and rocky stagnant pools [RSP, *n* = 1, in maroon]) on the Isle of May in Scotland, with known chlamydial species in the NCBI public database. Chlamydial Families are indicated to the right of the figure. Note there was no partitioning between live or dead seal with respect to the CLO species DNA recovered and all sediment samples contained *Rhabdochlamydiaceae* DNA which was present in nasal swabs from only six grey seal pups

All CLO DNA positive nasal swab samples, except ten, grouped closely with members of the *Parachlamydiaceae* cluster following BLAST analysis (Fig. 2). The remaining sequences grouped into three clusters (Fig. 2); three most closely with the *Simkaniaceae* (CD21, CL49 and CL66) and six with the *Rhabdochlamydiaceae* (CD15, CL16, CD17, CD41, CD49 and CL60), which also contained all of the seven positive sediment samples. The remaining sample, nasal swab CD37, appeared distinct compared to all the other samples and grouped most closely with the *Candidatus* (*Ca*.) Clavichlamydiaceae and *Ca.* Amphibiichlamydiaceae families, which are associated with CLO found in aquatic species [9, 21].

## Discussion

To our knowledge, this is the first report of *Chlamydia*-like organisms in any marine mammal, perhaps surprising given the abundant and diverse nature of Chlamydiae present in the environment [22, 23], marine sediment [24], marine fish that grey seals are known to eat [5, 8, 9, 25], freshwater turtles [26] and also livestock [15, 17] with inevitable run-off from agricultural land into coastal waters [1]. Given that grey seals spend most of their lives at sea, but come ashore and form dense colonies, principally to moult, breed and give birth [27], the potential for CLO to circulate between them and their environment during these times may signify an important but, as yet, unrecognized mode of transmission and dissemination for these bacteria. However, as the sampling site for this work was an island 8 km offshore from the Scottish mainland and the animals sampled were pre-weaned pups that had not been to sea, this raises questions of how the CLO were transported to and maintained on an island devoid of livestock and where two of the main sites (Tarbet Slope and Rona Rocks in Fig. 1) are sufficiently far from the sea that they are well above the highest tidal zone.

*Rhabdochlamydiaceae* accounted for the DNA of CLO in all the sediment samples and are frequently associated with arthropods [28–32] which are abundant on the island due to nesting seabirds, which carry *Ixodes uriae*, and there is an abundance of rabbits and mice that would maintain any *Ixodes ricinus* introduced when livestock were on the island during the 19th and early 20th Centuries. Nasal swabs from six grey seal pups contained *Rhabdochlamydiaceae* DNA suggesting the latter may simply be environmental contamination due to inhalation of, or nasal immersion by the pup in, the underlying substrate. DNA sequences from a further three samples (CL66, CL49 and CD21) formed a distinct clade, clustering with both *Rhabdochlamydiaceae* and *Simkaniaceae* (genus *Ca*. Syngnamydia) species, the latter also being associated with epitheliocystis in fish [33–35], and suggesting increased diversity and perhaps an as yet undefined new family of related organisms.

The majority of the sequences obtained from seal pups with nasal swabs positive for CLO clustered most closely with *Parachlamydiaceae* species, forming separate and distinct clades perhaps representing new diversity and new family lineages. However, these sequences were distinct from those obtained from the limited number of sediment samples, suggesting that they could be commensal in the upper respiratory tract of grey seals. *Parachlamydia* species were initially described as endosymbionts of *Acanthamoeba* [11]. They have been identified in amoeba derived from a diverse range of sources, including sewage sludge, water treatment plants, fresh and salt water environments [16, 23], as well as being successfully isolated from them [12, 36]. It is this resistance to digestion by amoeba that provides a possible aquatic reservoir for transmission of CLO to a variety of aquatic species. Therefore, it would not be unreasonable to speculate that transmission to the adult seals is via this route direct from sea water or via their diet. Furthermore, we have previously shown that DNA from these organisms can be detected in bulk milk tank samples taken from dairy cows [13], so this could also then suggest that milk is a possible natural route of transmission from adults to the pre-weaned pups, but this would require investigation.

Although CLO species from the *Parachlamydiaceae* family are ubiquitous in both fresh water and marine environments, they are also known to be pathogenic causing human adult and neonatal respiratory disease [5]. Additionally, it has been suggested they have the capacity to cross the human placenta and infect the foetus [5] and are proposed abortifacients in cattle [15, 17]. The pathogenicity in seals remains unknown despite comprehensive necropsies carried out on the dead seals sampled in this study for which the cause of death was attributed to starvation, omphalitis-peritonitis, septicaemia, stillbirth and trauma [19]. In this study, the lack of difference in CLO DNA recovery rates from live versus dead seals suggests they are commensal, although *Parachlamydia* spp. may have the potential to be opportunistic pathogens in conjunction with co-factors such as environmental stressors, exposure to pollutants, paucity of food and/or as a result of co-infection with other known pathogens.

*Chlamydiaceae* family members of the *Chlamydiales* order are well established pathogens causing disease in their human and animal hosts [37], including fish [5, 8]. *Ca.* Piscichlamydia salmonis and *Ca.* Clavichlamydia salmonicola are now regarded as etiological agents in complex gill disease in both salt and fresh water farmed salmonids [5, 8, 25]. Given the above, it is possible that such pathogenic potential could also be extended to the novel *Parachlamydiales* species identified through this work. Therefore, the relatively high frequency of CLO in the nasal cavities of grey seal pups justifies further investigation in cases of respiratory and reproductive diseases. However, such investigations would need to be targeted, due to the specific requirements for the recovery and identification of these organisms, as they will not be detected using the standard diagnostic bacteriological techniques currently employed by EU based marine mammal stranding schemes [38].

As humans and animals share health risks from changing environments, further studies are warranted to improve our understanding of the incidence and pathogenic potential of these organisms in both humans and in sentinel animals such as the grey seals in this study. Even more so, as the latter are exposed to marine and coastal environments directly, and to agricultural and urban locations due to run-off through watercourses and discharge of resultant effluent from sewage treatment plants. Such studies should include further molecular epidemiological evaluation, as well as detailed histologic/immunohistochemical investigation of archival and prospective tissue samples to determine if these organisms are lesion-associated. To determine any disease causation, it will be important to recover live organisms using established amoebal co-culture techniques and conduct specific challenge studies.

## Conclusions

This study has identified the presence of 16S rDNA of CLOs on nasal swabs taken from pre-weaned grey seal pups, as well as from their natal substrates. The sequences clustered with three *Parachlamydiaceae* families that are found in other aquatic species and which have been associated with various pathologies. The identification of CLO DNA in these animals before they go to sea indicates a probable direct acquisition from the mothers, diet or natal substrate, and may be indicative of a wide occurrence of these organisms in both aquatic and land environments resulting from environmental pollution due to anthropogenic activities such as sewage discharge and agricultural run-off. However, grey seals from the Isle of May forage over large areas visiting the coastal communities of other countries where they could disseminate CLO from the UK or acquire those originating in other countries.

## Methods

### Sample Collection

Between October and December 2011, nasal swabs (346C, Sterilin, Newport, UK) were taken from live (CL, *n* = 42) and dead (CD, *n* = 50) pups, approximately 28 days old on average, along with sediment samples (*n* = 8) from three main sites on the island populated by the breeding seals and where the CL pups were located (Fig. 1). The sediment samples were taken from three very different substrates: tidal boulder beach (TBB, *n* = 3) at Pilgrim’s Haven (56.1815° N 2.5511° W); muddy grassy slope (MGS, *n* = 3) at Tarbet Slope (56.19° N 2.5621° W); and rocky stagnant pools (RSP, *n* = 2) at Rona Rocks (56.1909° N 2.5618° W). Swabs were placed immediately into universal transport medium (UTM, Sterilin, Newport, UK) for transport back to the laboratory, stored initially at 4 °C and frozen at −80 °C within 12 h of collection for downstream processing and analysis.

### Sample extraction

Thawed swabs were placed in a sonicating bath for 30s and then centrifuged at 2000 x *g* for 10 min at 4 °C. Nucleic acids were extracted from nasal swab supernatants using a NorDiagViral NA Arrow automated extraction robot (Isogen Life Science, De Meern, Netherlands) and from sediment samples using a Power Soil DNA isolation kit (Qiagen, Manchester, UK), in accordance with manufacturers’ instructions.

### PCR, cloning and sequencing

A Pan-*Chlamydiales*/*Parachlamydiales* ‘touch down’ PCR targeting the 16S rRNA gene was performed using primers CHL16SFOR2 [5′-GTGGATGAGGCATGCAAGTCGA-3′] and CHL16SREV2 [5′-CAATCTCTCAATCCGCCTAGACGTCTTAG-3′], as previously described [39], to generating amplicons of approximately 260 bp. Negative-control reactions contained DNA-free water in place of extracted DNA. Each reaction included 25 μl of PCR BioMix buffer (BIO-25012, London UK), 5 μl of extracted DNA and 2.5 μl of each primer (0.5 pmol final concentration) made up to a final volume of 50 μl in water. Amplified products were electrophoresed on a 2% agarose gel, gel purified (GeneJET PCR purification Kit, Thermo Fisher Scientific, Loughborough, UK), ligated into general cloning vector pGEMT-Easy (Promega UK Ltd., Southampton, UK) and transformed into NEB 5-alpha Competent Subcloning Efficiency *E. coli* cells (New England BioLabs, Hitchin, UK). Successfully transformed cells were selected by ampicillin resistance and blue–white colony selection, according to standard procedures. The insertion of PCR products (as determined by an increase in fragment size following gel electrophoresis) was confirmed by colony PCR using universal sequencing primers T7 and SP6. Plasmid DNA was prepared from each clone (QIAprep Spin Miniprep Kit, Qiagen, UK) and sequenced on an ABI 3730xl DNA Analyzer (MWG Operon, Ebersberg, Germany) using T7 and SP6 universal sequencing primers.

### Sequence analysis

For each sample which yielded sequence information, paired reads were trimmed (DNASTAR Lasergene SeqMan Pro software, DNASTAR, Inc., USA) to generate a single consensus sequence and aligned to a representative set of 45 *Chlamydiales/Parachlamydiales* 16S rDNA sequences identified by BLAST analysis; plus an out-group of eight non-*Chlamydiales* sequences (broad representatives of the bacterial kingdom including the closely related *Verrucomicrobia* species) (Fig. 2). Using RFold as the structure predictor [40] and R-Coffee Instance for alignment [41], a PhyML inference tree was estimated (midpoint rooted tree; settings: substitution model general time reversible + gamma (GTR + G); 100 bootstrap runs) using the PhyML program launched from the TOPALi v2 package [42].

### Statistical analyses

Rates of recovery of CLO DNA from nasal swabs between CL and CD seal pups were compared by the Chi-squared test with Yates’ continuity correction using R version 3.6.3 [20]. Statistical significance was set at p ≤ 0.05.

## Data Availability

The datasets generated and analysed during the current study are available in the GenBank repository under accession numbers KT258813-KT258870.

## Abbreviations

CLO: *Chlamydia*-like organisms
CL: Samples from live seal pups
CD: Samples from dead seal pups
TBB: Sediment samples from tidal boulder beach at Pilgim’s Haven
MGS: Sediment samples from muddy grass slope area at Tarbet Slope
RSP: Sediment samples from rocky stagnant pools at Rona Rocks
